# Mid-Point of the Active Phase Is Better to Achieve the Natriuretic Effect of Acute Salt Load in Mice

**DOI:** 10.3390/nu15071679

**Published:** 2023-03-30

**Authors:** Momoko Imamura, Hiroyuki Sasaki, Katsuki Hayashi, Shigenobu Shibata

**Affiliations:** Laboratory of Physiology and Pharmacology, School of Advanced Science and Engineering, Waseda University, Shinjuku-ku, Tokyo 162-8480, Japan; momoko_imamura@ruri.waseda.jp (M.I.); hiroyuki-sasaki@aoni.waseda.jp (H.S.); tx1y3iz@ruri.waseda.jp (K.H.)

**Keywords:** potassium, sodium, urinary excretion, circadian rhythm, intake timing

## Abstract

Excess sodium intake and insufficient potassium intake are a prominent global issue because of their influence on high blood pressure. Supplementation of potassium induces kaliuresis and natriuresis, which partially explains its antihypertensive effect. Balancing of minerals takes place in the kidney and is controlled by the circadian clock; in fact, various renal functions exhibit circadian rhythms. In our previous research, higher intake of potassium at lunch time was negatively associated with blood pressure, suggesting the importance of timing for sodium and potassium intake. However, the effects of intake timing on urinary excretion remain unclear. In this study, we investigated the effect of 24 h urinary sodium and potassium excretion after acute sodium and potassium load with different timings in mice. Compared to other timings, the middle of the active phase resulted in higher urinary sodium and potassium excretion. In *Clock* mutant mice, in which the circadian clock is genetically disrupted, urinary excretion differences from intake timings were not observed. Restricted feeding during the inactive phase reversed the excretion timing difference, suggesting that a feeding-induced signal may cause this timing difference. Our results indicate that salt intake timing is important for urinary sodium and potassium excretion and provide new perspectives regarding hypertension prevention.

## 1. Introduction

Excess sodium intake and insufficient potassium intake result in high blood pressure (BP), and their association with the risk of cardiovascular diseases (CVDs) has been confirmed [1,2]. The World Health Organization (WHO) aims to reduce peoples’ Na intake and increase their K intake [3], and many campaigns have been conducted to meet these goals. However, there is still a discrepancy between the recommended and actual intake of Na^+^ and K^+^. According to the National Nutrition Survey in Japan (NNSJ) in 2019, the average Na^+^ intake in the Japanese population was higher than the recommended intake, whereas the average K^+^ intake was lower [4].

Supplementation of K^+^ has shown antihypertensive effects in both humans and animals [5,6]. An acute K^+^ load sustained both kaliuresis and natriuresis, and the natriuretic effect of K^+^ explains its BP-lowering effects [7]. The natriuretic effect is partly explained by the inhibition of renal Na^+^ reabsorption, in which rapid dephosphorylation of Na^+^/Cl^−^ cotransporter (NCC) occurs in the distal convoluted tubule (DCT). Therefore, NCC, a key molecule for regulating urinary K^+^ excretion may be involved [8]. The activation of transporters, such as the renal outer medullary potassium channel and epithelial Na^+^ channel (ENaC), is also considered to play a role [7].

The kidneys, where electrolyte balancing occurs, are known to be controlled by the circadian clock [9]. The master clock in the suprachiasmatic nucleus (SCN) organizes peripheral tissue clocks, such as the kidney clock [10]. Molecular clocks in all cells share the same mechanism [11]. A heterodimer of CLOCK and BMAL1 proteins binds to a region of DNA called the E-box located upstream of the *Per* and *Cry* genes, promoting the transcription of *Per* and *Cry*. PER and CRY proteins are translated in the cytoplasm, form a dimer, and inhibit the transcription of the heterodimer of CLOCK and BMAL1 proteins, thereby suppressing the transcription of *Per* and *Cry*. The feedback loop of transcription and translation forms the rhythm of the circadian clock which occurs over approximately 24 h and affects various functions [12]. BP peaks in the morning and dips at night [13]. Urinary Na^+^ and K^+^ excretion, as well as regulators of water–sodium balance (V2R, Aqp2, Aqp4, αENaC), exhibit circadian rhythms [14,15]. *Clock* mutant mice show increased urinary Na^+^ excretion and urine volume during the day [14,16]. Urinary Na^+^ excretion rhythms of humans are observed as well, reaching a maximum during the day and minimum at night, positively correlating with BP over 24 h [17]. The secretion of aldosterone, the mineralocorticoid hormone that stimulates Na^+^ reabsorption, is greater during the day than at night, with a clear peak in the morning [9].

Since renal function exhibits circadian rhythms, the response to Na^+^ and K^+^ intake may change depending on the timing of intake. Food intake timing is an important regulator of the peripheral clock, and restricted feeding (RF) has been shown to entrain the peripheral clock [18]. RF entrains the liver clock, with or without the SCN [19]. Tahara et al. showed that 12 h of RF at night could synchronize the peripheral clocks of SCN-lesioned mice, including kidney function [20]. Ikeda et al., revealed that, while adrenalectomy alone did not affect clocks in mice, feeding six meals per day to reduce feeding-induced signals caused phase advances, and adrenalectomy and six-meals RF together caused impairment of the kidney and liver clocks, suggesting that both feeding and adrenal entrainment stimuli are necessary for these clocks to oscillate normally [21].

In our previous study, the sodium-to-potassium ratio at lunchtime was positively associated with BP, and K^+^ intake was negatively associated with BP, whereas this association was not observed at breakfast or dinner time, suggesting that the intake of K^+^ for the prevention of high BP may be time-dependent [22]. Johnston et al. showed that natriuretic response to acute Na^+^ load is not different at zeitgeber time 0 (ZT0) or zeitgeber time 12 (ZT12) in normal rats; however, endothelin B receptor-deficient male rats showed a delayed natriuretic response at ZT12 [23]. Various studies have shown that nutrients have greater health benefits at different times of the day [24]. The effects of the timing of Na^+^ and K^+^ intake remain unknown. Therefore, in this study, we first investigated the effects of different timing of Na^+^ and K^+^ intake at four different time points (morning, noon, evening, and midnight) on urinary excretion in mice. Next, using *Clock* mutant mice, in which the circadian clock is genetically disrupted [16], we examined whether the effects of Na^+^ and K^+^ intake differ without the circadian clock. Finally, we conducted time-restricted feeding to determine the effect of feeding-induced rhythm on the intake timing of Na^+^ and K^+^.

## 2. Materials and Methods

### 2.1. Animals and Experimental Diets

All animal care procedures followed the guidelines of the Committee for Animal Experimentation of the School of Science and Engineering at Waseda University and those prescribed by the Japanese government. The experiments were approved by the School of Science and Engineering of Waseda University (Approval number: A22-066 and approved date: 28 March 2022). For this study, male ICR mice and homozygous *Clock^∆19/^^∆19^* mutant mice on ICR background aged 8–10 weeks old were used, as previously confirmed [25]. In the current experiments, we did not check the arrhythmicity of *Clock* mutant mice except for Na^+^ and K^+^ urinary excretion. However, we already checked the abnormality of locomotor activity, blood pressure and heart rate in our recent paper [16]. Mice were housed and maintained under a 12 h light/12 h dark cycle (with lights on at 8:00 AM and lights off at 8:00 PM) at room temperature (23 °C ± 2 °C) and 60% ± 5% humidity. Lights-on time was defined as zeitgeber time 0 (ZT0), and light-off time was defined as zeitgeber time 12 (ZT12).

Mice were fed a normal diet (MF; Oriental Yeast Co., Ltd., Tokyo, Japan) which contains 0.6% NaCl unless stated otherwise. Mice on a high-salt diet (1.6% NaCl) were fed MF supplemented with 1.0% NaCl (Oriental Yeast Co., Ltd., Tokyo, Japan). Both diets, normal diet (MF) and high-salt diet (HSD), contain 0.9% potassium. All the mice had free access to water during the experiment. During experiments with time-restricted feeding, the active period feeding group (AF) could access food from ZT12 to ZT0 during the active phase. The inactive period feeding group (IF) could access food from ZT0 to ZT12 during the inactive phase. Mice were kept under a feeding schedule for at least two weeks before urine collection.

### 2.2. Metabolic Cage

The mice were individually housed in metabolic cages (Shinano Corporation, Tokyo, Japan) for urine collection. Water intake was measured, and urine and feces were collected continuously for one day (24 h). Before the experiments, the mice were placed in metabolic cages for three days for acclimation and one day of recovery. Urine samples were kept at −20 °C until analysis.

### 2.3. Experimental Design

**Figure 1:**
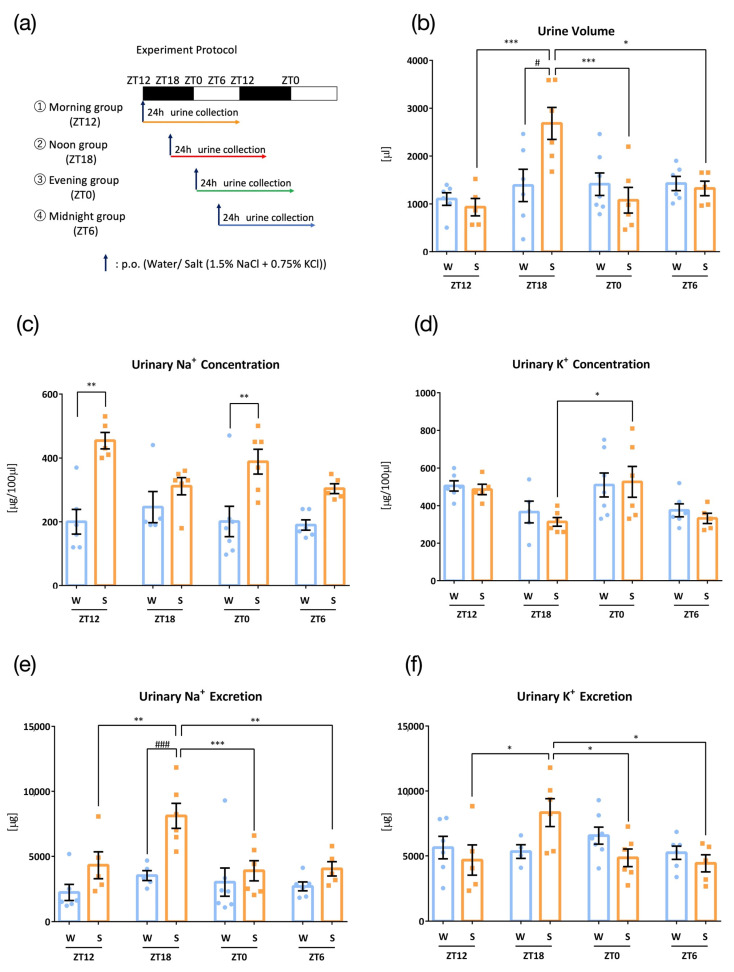
Urinary excretion after administration of salt load at four time points (W: Water administration, S: Salt administration). (**a**) Experimental protocol, (**b**) Urine volume, * *p* < 0.05, *** *p* < 0.001 (vs. zeitgeber time 18 (ZT18)), # *p* < 0.05 (vs. water) Tukey. (**c**) Urinary Na^+^ concentration, ** *p* < 0.01 (vs. ZT6) Benjamini, Krieger, and Yekutieli’s two-stage test. (**d**) Urinary K^+^ concentration, * *p* < 0.05 (vs. ZT18) Tukey. (**e**) Urinary Na^+^ excretion, ** *p* < 0.01, *** *p* < 0.001 (vs. ZT18), ### *p* < 0.001 (vs. water) Tukey. (**f**) Urinary K^+^ excretion, * *p* < 0.05 (vs. ZT18). Data are represented as mean ± SEM (ZT12 group: *n* = 5–6, ZT18 group: *n* = 7–8, ZT0 group: *n* = 6–7, ZT6 group: *n* = 5–6).

In the first experiment, mice were divided into four groups depending on the administration time points (ZT12 (the start of the active phase), ZT18 (the midpoint of the active phase), ZT0 (the start of the inactive phase), and ZT6 (the midpoint of the inactive phase)) to test whether urinary Na^+^ and K^+^ excretion varied at different time points (Figure 1a). After 3 days of acclimatization to the metabolic cage environment, and one day of recovery, mice in the four groups were orally administered 0.1 mL/10 g (Body Weight, BW) of water at ZT12, ZT18, ZT0 and ZT6 and housed in metabolic cages for one day, and 24-h urine was collected. Next, mice in the four groups were orally administered 0.1 mL/10 g (BW) of salt (1.5% NaCl + 0.75% KCl) at each time point, and 24 h urine was collected.

**Figure 2:**
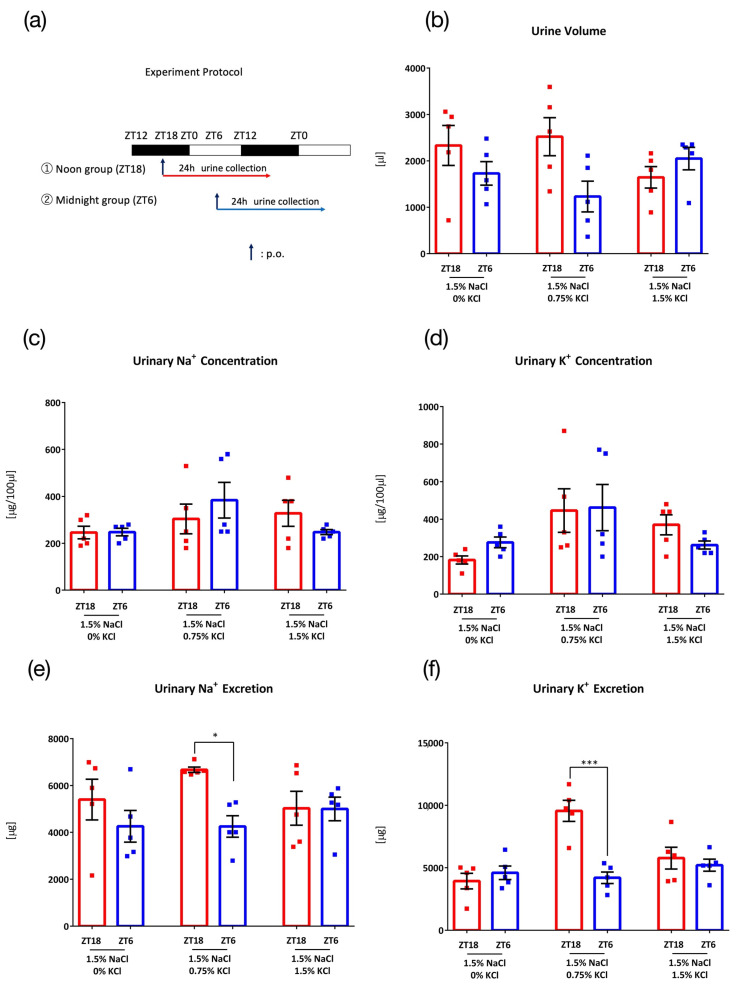
Urinary excretion after administration of salt load of different KCl concentrations. (**a**) Experimental protocol, (**b**) Urine volume. (**c**) Urinary Na^+^ concentration, (**d**) Urinary K^+^ concentration, (**e**) Urinary Na^+^ excretion, * *p* < 0.05 (vs. ZT6) Benjamini, Krieger and Yekutieli’s Two-stage. (**f**) Urinary K^+^ excretion, *** *p* < 0.001 (vs. ZT6) Sidak. Data are represented as mean ± SEM (ZT18 group: *n* = 5, ZT6 group: *n* = 5).

In the second experiment, mice were divided into two groups depending on the administration time points (ZT18 and ZT6) and administered different concentrations of salt (1.5% NaCl + 0% KCl, 1.5% NaCl + 0.75% KCl, and 1.5% NaCl + 1.5% KCl) to determine the effect of different potassium concentrations on urinary excretion (Figure 2a). After 3 days of acclimatization to the metabolic cage environment, and one day of recovery, mice in the two groups were orally administered 0.1 mL/10 g (BW) of salt (1.5% NaCl + 0% KCl) at ZT18 or ZT6 and housed in metabolic cages for one day, and 24 h urine was collected. The same protocol was repeated with 1.5% NaCl + 0.75% KCl, and 1.5% NaCl + 1.5% KCl and 24 h urine was collected.

**Figure 3:**
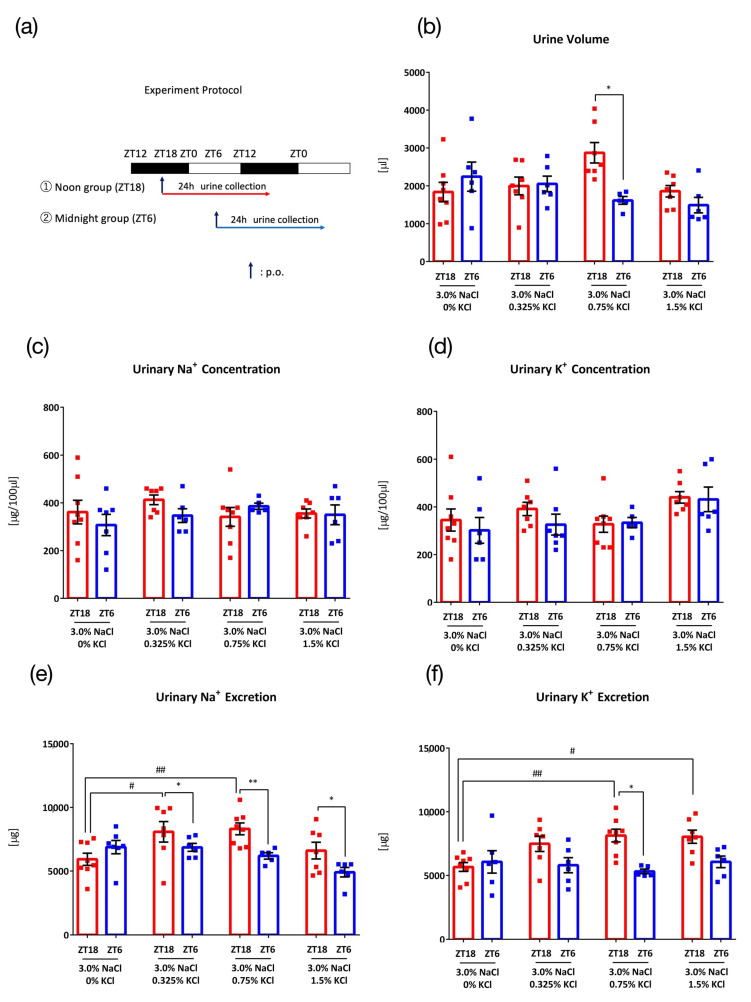
Urinary excretion after administration of salt load of different KCl concentrations against 3.0% NaCl. (**a**) Experimental protocol, (**b**) Urine volume, * *p* < 0.05 (vs. ZT6) Sidak. (**c**) Urinary Na^+^ concentration, (**d**) Urinary K^+^ concentration, (**e**) Urinary Na^+^ excretion, * *p* < 0.05, ** *p* < 0.01 (vs. ZT6), # *p* < 0.05, ## *p* < 0.01 (vs. 3.0% NaCl, 0% KCl) Benjamini, Krieger and Yekutieli’s Two-stage. (**f**) Urinary K^+^ excretion, * *p* < 0.05 (vs. ZT6), # *p* < 0.05, ## *p* < 0.01 (vs. 3.0% NaCl, 0% KCl) Sidak. Data are represented as mean ± SEM (ZT18 group: *n* = 7–8, ZT6 group: *n* = 5–6).

In the third experiment, mice were divided into two groups depending on the administration time points (ZT18 and ZT6) and administered different concentrations of KCl (0%, 0.325%, 0.75%, and 1.5%) against 3.0% NaCl, a higher concentration of NaCl to determine the appropriate KCl concentration to accelerate Na^+^ excretion in the higher NaCl concentration condition (Figure 3a). After 3 days of acclimatization to the metabolic cage environment, and one day of recovery, mice in the two groups were orally administered 0.1 mL/10 g (BW) of salt (3.0% NaCl + 0% KCl) at ZT18 or ZT6 and housed in metabolic cages for one day, and 24 h urine was collected. The same protocol was repeated with 3.0% NaCl + 0.325% KCl, 3.0% NaCl + 0.75% KCl, and 3.0% NaCl + 1.5% KCl and 24 h urine was collected.

**Figure 4:**
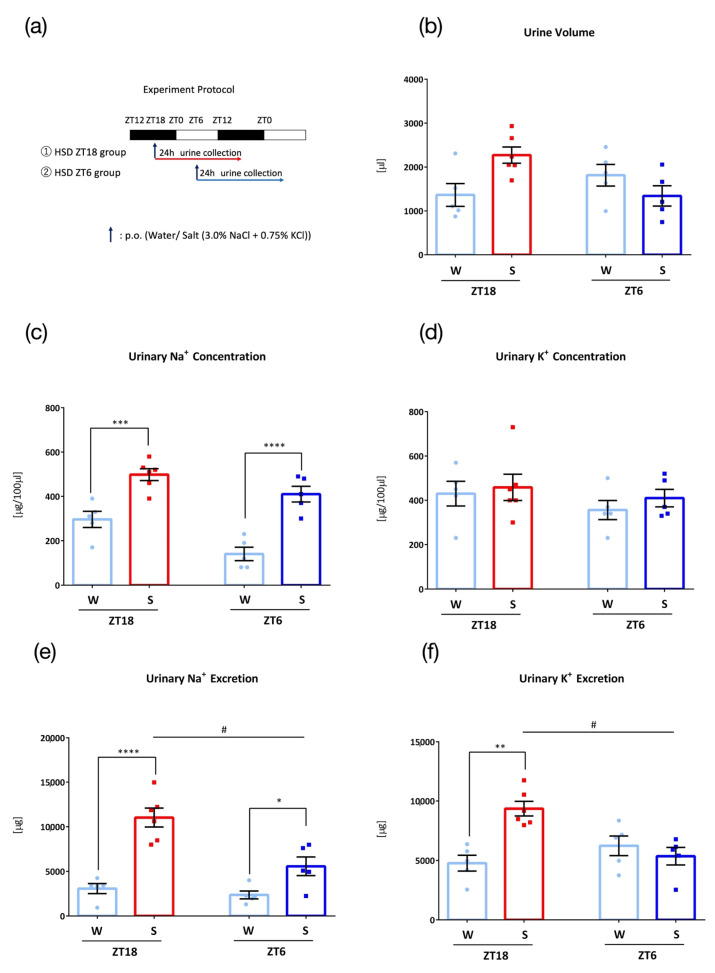
Urinary excretion after administration of salt load with a high-salt diet (W: Water administration, S: Salt administration). (**a**) Experimental protocol, (**b**) Urine volume, (**c**) Urinary Na^+^ concentration, *** *p* < 0.001, **** *p* < 0.0001 (vs. Water) Sidak. (**d**) Urinary K^+^ concentration, (**e**) Urinary Na^+^ excretion, # *p* < 0.01 (vs. ZT18), * *p* < 0.05, **** *p* < 0.0001 (vs. Water) Sidak. (**f**) Urinary K^+^ excretion, # *p* < 0.01 (vs. ZT18), ** *p* < 0.01 (vs. Water) Sidak. Data are represented as mean ± SEM (ZT18 group: *n* = 5–6, ZT6 group: *n* = 5).

In the fourth experiment, mice were divided into two groups depending on the administration time points (ZT18 and ZT6), and both groups were fed a high salt diet (HSD) for four weeks prior to the administration of salt and urine collection to examine the effect of chronic excessive salt intake on the timing difference of urine excretion (Figure 4a). After 3 days of acclimatization to the metabolic cage environment and one day of recovery, mice in the two groups were orally administered 0.1 mL/10 g (BW) of water at ZT18 or ZT6 and housed in metabolic cages for one day, and 24 h urine was collected. Next, mice in the two groups were orally administered 0.1 mL/10 g (BW) of salt (3.0% NaCl + 0.75% KCl) at each time point, and 24 h urine was collected.

**Figure 5:**
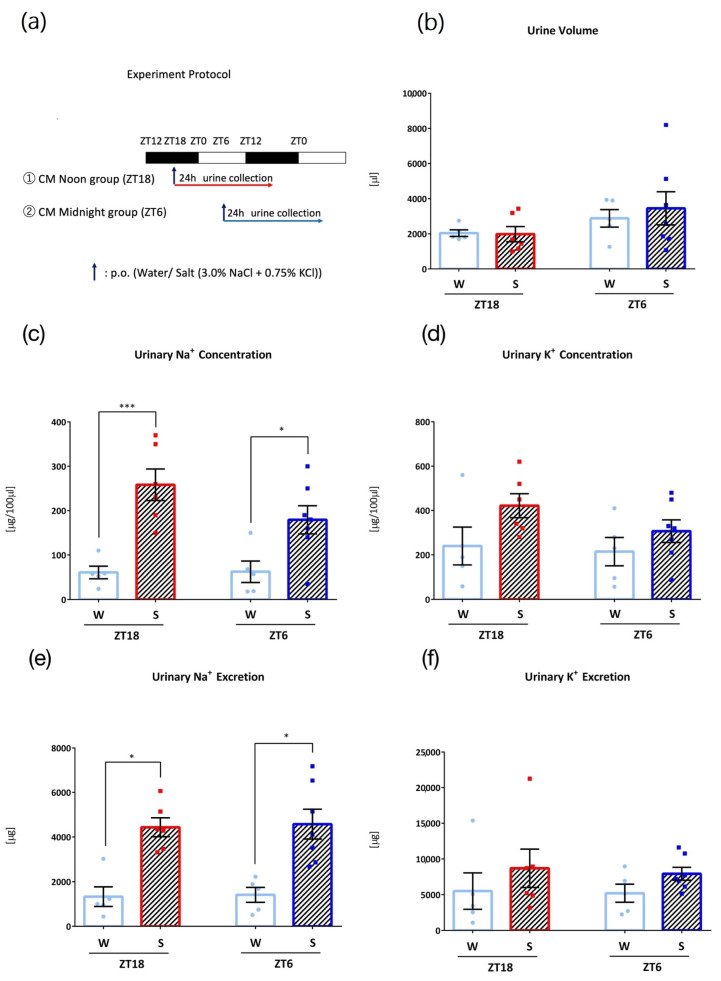
Urinary excretion after administration of salt load in Clock mutant (CM) mice (W: Water administration, S: Salt administration). (**a**) Experimental protocol, (**b**) Urine volume, (**c**) Urinary Na^+^ concentration, * *p* < 0.05, *** *p* < 0.001 (vs. Water) Sidak. (**d**) Urinary K^+^ concentration, (**e**) Urinary Na^+^ excretion, * *p* < 0.05 (vs. Water) Sidak. (**f**) Urinary K^+^ excretion. Data are represented as mean ± SEM (ZT18 group: *n* = 5–6, ZT6 group: *n* = 5–6).

In the fifth experiment, *Clock* mutant mice were divided into two groups depending on the administration time points (ZT18 and ZT6) to examine the role of the circadian clock (Figure 5a). After 3 days of acclimatization to the metabolic cage environment and one day of recovery, mice in the two groups were orally administered 0.1 mL/10 g (BW) of water at ZT18 or ZT6 and housed in metabolic cages for one day, and 24 h urine was collected. Next, mice in the two groups were orally administered 0.1 mL/10 g (BW) of salt (3.0% NaCl + 0.75% KCl) at each time point and 24 h urine was collected.

**Figure 6:**
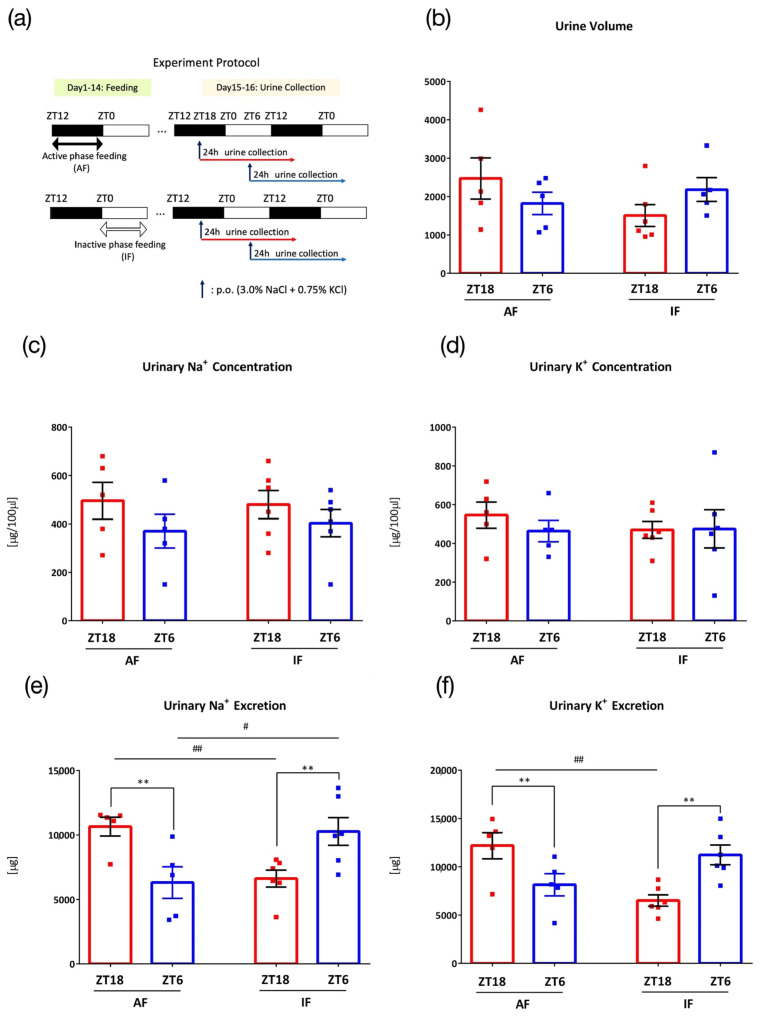
Urinary excretion after administration of salt load under time-restricted feeding. (**a**) Experimental protocol, (**b**) Urine volume, (**c**) Urinary Na^+^ concentration, (**d**) Urinary K^+^ concentration, (**e**) Urinary Na^+^ excretion, # *p* < 0.05, ## *p* < 0.01 (vs. the inactive period feeding group (IF)), ** *p* < 0.01 (vs. ZT6) Benjamini, Krieger and Yekutieli’s two-stage. (**f**) Urinary K^+^ excretion, ## *p* < 0.01 (vs. IF), ** *p* < 0.01 (vs. ZT6) Sidak. Data are represented as mean ± SEM (AF group: *n* = 5, IF group: *n* = 6). Male ICR mice were used for this experiment.

In the sixth experiment, mice were under a time restricted feeding condition to examine the effect of eating time on urine excretion rhythm. Mice were divided into an active period feeding group (AF), which could access food from ZT12 to ZT0 during the active phase, and an inactive period feeding group (IF), which could access food from ZT0 to ZT12 during the inactive period. Both groups were under the time-restricted feeding schedule for 2 weeks. After 3 days of acclimatization to the metabolic cage environment and one day of recovery, mice in both groups were orally administered 0.1 mL/10 g (BW) of salt (3.0% NaCl+ 0.75% KCl) at ZT18 or ZT6 and housed in metabolic cages for 1 day, and 24 h urine was collected (Figure 6a).

### 2.4. Urinary Measurements

Urine Na^+^ and K^+^ concentrations were determined using an ion meter (HORIBA, Kyoto, Japan). The amounts of urinary Na^+^ and K^+^ were obtained by calculation of [urine volume × each Na^+^ and K^+^ concentration].

### 2.5. Statistical Analysis

Statistical analysis of the data was performed using GraphPad Prism version 9.1.1 (GraphPad Software, San Diego, CA, USA). The data were divided into parametric and non-parametric tests by analyzing the normal distribution and variance. Normal distribution was examined using the D’Agostino–Pearson test/Kolmogorov–Smirnov test, and variance was examined using the F-value test/Bartlett’s test. If the data were parametric, statistical significance was determined using Student’s *t*-test or two-way ANOVA with a Sidak test (if the interaction was not significant, but the main effect was significant), or Tukey test (if the interaction was significant) for post-hoc analysis. If the data were non-parametric, statistical significance was determined using the Kruskal–Wallis test with Benjamini, Krieger, and Yekutieli’s two-stage test. All data are expressed as the mean ± standard error of the mean (SEM), and *p* < 0.05 was considered statistically significant.

## 3. Results

### 3.1. Comparison of Four Intake Timings

In the first experiment, mice were administrated water or salt (1.5% NaCl + 0.75% KCl) at different time points (ZT12, ZT18, ZT0, and ZT6), and 24 h urine was collected to test whether urinary Na^+^ and K^+^ excretion varied at different time points (Figure 1a). Urine volumes did not vary at different time points with administration of water; however, salt load resulted in higher urine volume at ZT18, the mid-point of the active phase of mice (Figure 1b). Urinary Na^+^ concentrations were higher with salt load than those with water load at ZT12 and ZT0 (Figure 1c). Urinary K^+^ concentrations did not differ between water and salt administration, and ZT0 showed higher urinary K^+^ concentrations than those seen at ZT18 (Figure 1d). Urinary Na^+^ and K^+^ excretion was also significantly higher at ZT18 than that at other times, with oral administration of salt, however, not with water (Figure 1e,f). Although urinary Na^+^ was higher in salt group than water group at ZT12 and ZT0 (Figure 1c), urinary excretion of Na^+^ was similar between water group and salt group. This is because of smaller urine volume in salt group compared to water group at ZT12 and ZT0. Thus, urine volume differences are easy to reflect the Na^+^ secretion.

### 3.2. Comparison of Salt Load with Different KCl Concentrations

To determine the effect of different KCl concentrations on urinary excretion, mice were administered 0.1 mL/10 g (BW) of 3 different concentrations of salt (1.5% NaCl + 0% KCl, 1.5% NaCl + 0.75% KCl, and 1.5% NaCl + 1.5% KCl) at ZT18 or ZT6 (Figure 2a). With 1.5% NaCl + 0% KCl, no timing difference between ZT18 and ZT6 was observed in the urine volume and urinary Na^+^ concentration, urinary K^+^ concentration, urinary Na^+^ excretion, or urinary K^+^ excretion (Figure 2b–f, left columns). With the administration of 1.5% NaCl + 0.75% KCl, higher urinary Na^+^ excretion and urinary K^+^ excretion were observed (Figure 2e,f, middle columns), confirming the result from experiment 1 (Figure 1e). However, in contrast to experiment 1, urinary volume was not significantly higher in ZT18 than in ZT6 (Figure 2b, middle columns). This may be explained by individual mouse differences and the small number of mice. When the same amount of KCl as NaCl was administered (1.5% NaCl + 1.5% KCl), no timing differences were observed in urine volume, urinary Na^+^ concentration, urinary K^+^ concentration, urinary Na^+^ excretion, or urinary K^+^ excretion (Figure 2b–f, right columns).

### 3.3. Comparison of Salt Load with Different KCl Concentrations against Higher NaCl Concentration

To determine the appropriate KCl concentration to accelerate Na^+^ excretion in higher NaCl concentration condition, four different concentrations of KCl (0%, 0.325%, 0.75%, and 1.5%) to 3.0% NaCl were orally administered 0.1 mL/10 g (BW) at ZT18 or ZT6 (Figure 3a). With 3.0% NaCl + 0% KCl, no timing difference between ZT18 and ZT6 was observed in the urine volume and urinary Na^+^ concentration, urinary K^+^ concentration, urinary Na^+^ excretion, or urinary K^+^ excretion (Figure 3b–f). When K^+^ was administered together with Na^+^, urinary Na^+^ excretion varied between ZT18 and ZT6, suggesting the importance of K^+^ in causing this difference in time points of excretion of Na^+^. Of all concentrations, 3.0% NaCl + 0.75% KCl showed the most significant variation in time of excretion and significantly higher Na^+^ and K^+^ excretion than that with the salt load without K^+^. The urine volume also varied with time upon administration of 3.0% NaCl + 0.75% KCl.

**Figure A1:**
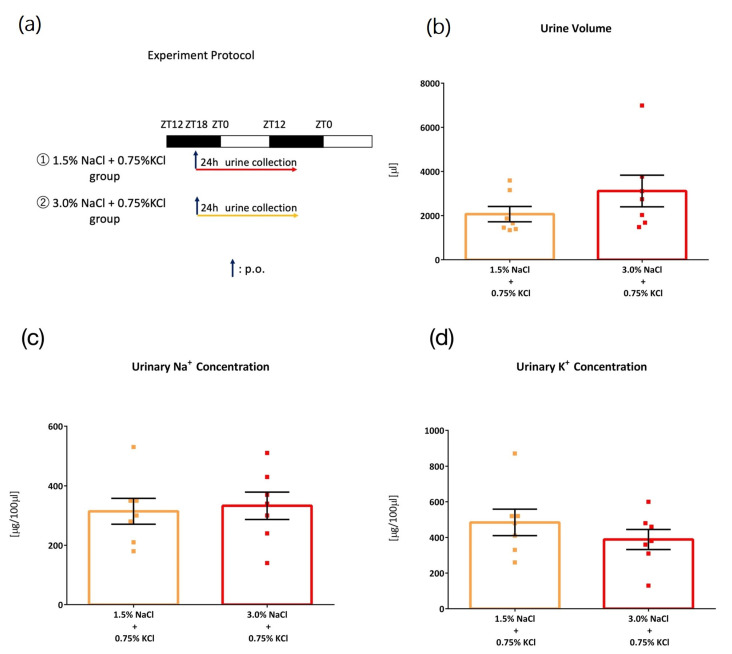

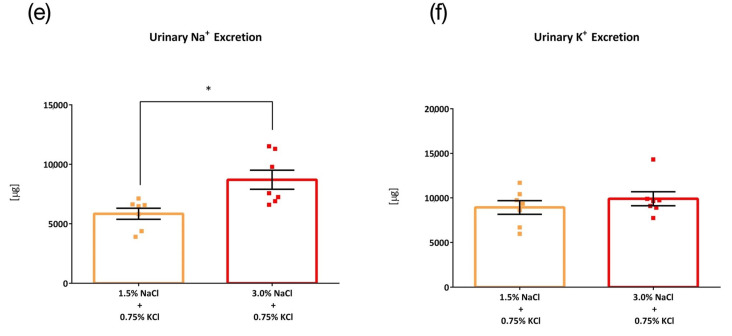
Urinary Excretion after administration of 1.5% NaCl + 0.75% KCl and 3.0% NaCl + 0.75% KCl. (**a**) Experimental Protocol, (**b**) Urine Volume, (**c**) Urinary Na^+^ Concentration, (**d**) Urinary K^+^ Excretion, (**e**) Urinary Na^+^ Excretion, * *p* < 0.05 (vs. 1.5% NaCl + 0.75% KCl) Student’s *t*-test. (**f**) Urinary K^+^ Excretion. Data are represented as mean ± SEM (1.5% NaCl + 0.75% KCl group: *n* = 7, 3.0% NaCl + 0.75% KCl group: *n* = 7).

Significant variation in time of excretion occurred with 0.75% KCl and both 1.5% NaCl and 3.0% NaCl (Figure 2e and Figure 3e). To determine the more effective dose of NaCl, we compared the effect of 3.0% NaCl and 1.5% NaCl on urinary Na^+^ excretion. As 3.0% NaCl exhibited higher urinary Na^+^ excretion than 1.5% NaCl, 3.0% NaCl was used as the experimental condition in the following experiments (Figure A1 in Appendix A).

### 3.4. Effects of High Salt Diet

To examine the effect of chronic excessive salt intake, mice were fed a high salt diet (HSD), with 1.0% NaCl added MF, for four weeks before 0.1 mL/10 g (BW) oral administration of water or salt (3.0% NaCl + 0.75% KCl) (Figure 4a). At both time points, a natriuretic effect was observed after the administration of salt load; however, administration at ZT18 showed significantly higher urinary sodium and potassium excretion compared to that observed at ZT6 (Figure 4b–f).

### 3.5. Effects of Intake Timing on Clock Mutant Mice

To examine the role of the circadian clock in the timing-dependent differences in Na^+^ and K^+^ excretion observed, an experiment was conducted with Clock mutant mice that followed a similar protocol, with 0.1 mL/10 g (BW) oral administration of water or salt (3.0% NaCl + 0.75% KCl) (Figure 5a). Clock mutant mice showed salt-induced Na^+^ excretion at both timings, and the timing difference in urinary Na^+^ or K^+^ excretion after salt load was not observed (Figure 5b–f).

### 3.6. Effects of Intake Timing on Mice under Time-Restricted Feeding

Time-restricted feeding can reset clock gene expression rhythms in peripheral organs, including the kidneys [20]. Therefore, time-restricted feeding at different clock times may affect the salt-load-induced Na^+^ and K^+^ excretion rhythms. Mice were divided into two groups: the active period feeding group (AF) and the inactive period feeding group (IF), which were kept under a feeding schedule for 2 weeks prior to urine collection, and the 24 h urine collection was conducted after the 0.1 mL/10 g (BW) administration of salt (3.0% NaCl + 0.75% KCl) (Figure 6a). While the AF group showed higher urinary Na^+^ and K^+^ excretion at ZT18 than at ZT6, the difference was reversed in the IF group, wherein urinary Na^+^ and K^+^ excretion was higher at ZT6 than that at ZT18 (Figure 6b–f).

## 4. Discussion

The main finding of our study is that urinary Na^+^ and K^+^ excretion changes according to the intake timing of Na^+^ and K^+^, with the mid-point of the active phase showing maximum excretion over the other timings. This difference was not observed with Na^+^ alone, suggesting the importance of the timing of K^+^ intake. Furthermore, this difference was reversed when the feeding pattern was reversed. Therefore, food intake rhythm may be a key factor in acute K^+^-induced natriuresis and kaliuresis.

Natriuresis and kaliuresis are induced upon administration of acute K^+^ load [26]. Aldosterone-independent dephosphorylation and inactivation of NCC occurs owing to acute K^+^ load in the DCT, and increased Na^+^ is delivered to the collecting duct, which exceeds the capacity for epithelial Na^+^ channel (ENaC)-mediated Na^+^ reabsorption, which is thought to cause natriuresis [6]. While the total amount of NCC (tNCC) remains unchanged during the day, phosphorylated NCC (pNCC) levels show diurnal rhythms that are aldosterone-dependent in the mouse kidney [27]. Layton and Gumz showed a time profile of circadian-regulated transporter activities; the study revealed that ENaC activity is the lowest during the middle of the active phase of mice [28]. Furthermore, in a model simulation, Gumz also showed that delivery of Na^+^ and K^+^ into the proximal tubules, loops of Henle and DCT, as well as the rate of urine excretion, are faster at ZT16 than that at the other time points in the day (ZT4, ZT10, ZT22). They also predicted higher Na^+^ excretion at this time point [28]. As stated, many of these complex kidney systems follow a diurnal rhythm, and although the definite mechanism is unknown, these rhythms may have resulted in higher urinary excretion of Na^+^ and K^+^ at ZT18. We previously reported that potassium intake during lunchtime in humans is negatively associated with BP [22]. Therefore, the higher natriuretic effect at lunchtime could explain the positive effect of lunchtime K^+^ on BP.

At ZT12 and ZT0, urine volumes were similar between water administration and salt administration, whereas urinary Na^+^ concentrations were higher with salt administration than with water administration. In previous studies, it has been presented that the increase in urinary Na^+^ excretion from sodium intake mainly results from higher urinary Na^+^ concentration rather than increase in urine volume [29]. In our experiment, KCl was administered together with NaCl and supplementation of KCl has been demonstrated to result in higher urine volume [30]. The circadian variation in response to acute K^+^ load may have resulted in increase in urine volume not being observed at ZT0 or ZT12, resulting in 24-h urinary Na^+^ excretion at ZT12 and ZT0 being similar between water and salt administration.

HSD also induced acute salt-load-induced natriuresis and kaliuresis at ZT18, and higher urinary Na^+^ excretion at ZT18 than to that at ZT6. Jensen et al. showed that HSD (2.0% NaCl) led to higher excretion of sodium than normal diet after KCl gavage [31]. A study in which rats were fed the same concentration of NaCl in the diet (normal diet 0.6%, HSD 1.6%) as in our experiments showed that, although alterations in renal clock gene functions and a desynchrony between the cortex and the inner medulla were observed, the urinary excretion patterns remained unchanged [32]. They also showed that the urinary sodium excretion of the HSD (2.0% NaCl) group significantly increased compared to that of the normal diet group. A higher concentration of NaCl may alter these results, as HSD has been shown to affect the peripheral clock. Oike et al., showed that feeding a high-salt diet (4.0% NaCl) led to changes in the mRNA expression of the clock genes *DBP* and *Bmal1* of the kidney, showing a phase advance [33]. Gizowsk and Bourque reported that the injection of hypertonic saline significantly phase-advanced the circadian locomotor activity of mice [34]. Furthermore, HSD consisting of 4.0% NaCl restricted to inactive phase eating impaired the urinary Na^+^ excretion rhythm whereas, normal diet did not [35]. Our HSD results, which included 1.6% NaCl, showed a more distinct difference in times of Na^+^ and K^+^ excretion than the normal diet; however, other studies, such as Oike et al. [33], used NaCl concentrations up to 4.0% NaCl, so the results may have changed with a phase advance. Furthermore, an increase in urine volume from salt administration was not seen with HSD. HSD has been shown to result in higher urine volume compared to normal diet during both the active phase and the inactive phase [36]. Urine volume may have increased from HSD, blunting the change in urine volume from salt intake.

Differences in urinary Na^+^ and K^+^ excretion based on the intake timing of Na^+^ and K^+^ were not observed in *Clock* mutant mice. Previously, Zuber et al. showed that in *clock* deficient mice, αENaC RNA expression was lower than that in wild-type mice at ZT2, and urinary Na^+^ excretion was increased at this time point [15]. ENaC activity is also reduced when *Per1* is lost or inhibited [37]. Nikolaeva et al. reported that in *Clock* mutant mice, the rhythm of plasma aldosterone levels is lost, and the 24-h mean aldosterone levels were not different from those in wild-type mice [14]. Furthermore, *Clock* mutant mice show a phenotype of nocturia and nocturnal polyuria, suggesting a role of *clock* in urine product rhythms [38]. Notably, circadian rhythms of transcripts that encode proteins involved in tubular reabsorption and secretion of various substrates, including Na^+^ and K^+^, have been reported to be lost in *Clock* mutant mice [14]. These disruptions in rhythms in *Clock* mutant mice may explain why higher urinary Na^+^ excretion at ZT18 compared to ZT6 was not seen in *Clock* mutant mice. Furthermore, abnormalities in clock genes have been suggested to result in lower urinary tract function [38]. For example, Negoro et al. reported the association of circadian changes in urinary bladder capacity in the regulation of clock genes, and that *Clock* mutant mice show a phenotype of nocturia and nocturnal polyuria [38,39]. The lower urinary tract function may have resulted in higher 24 h urine volume from water administration and may explain why the increase in urine volume from salt administration was not observed.

In the current experiment, no significant urinary Na^+^ or K^+^ concentrations (Figure 2c,d, Figure 3c,d, Figure 4d and Figure 6d) were observed among groups, but significant urinary Na^+^ or K^+^ excretions were observed among groups. Contrary, significant urinary Na^+^ concentrations (Figure 1c) were observed between water and salt groups, but no significant Na^+^ excretions were observed (Figure 1e). These results suggest the importance of urine volumes. Thus, to understand kidney Na^+^ and K^+^ excretion function, total urine volume may be critical information rather than information on spot urine volume and Na^+^ and K^+^ concentrations.

Feeding in the inactive phase reversed the difference in times of urinary sodium and potassium excretion, suggesting that the timing of food intake is important for natriuresis and kaliuresis. Generally, the kidney clock is endogenous [9]. However, several reports have shown the effects of time-restricted feeding on kidneys and BP. Zhang et al. reported that feeding restricted to the inactive phase led to reversed BP rhythm in mice [40]. Rhoads et al. reported that five days of time-restricted feeding led to a loss of plasma aldosterone rhythm in rats [35]. In both reports, administration of inactive time-restricted feeding consisting of a normal diet did not change the urinary Na^+^ excretion rhythm. The urinary K^+^ rhythm, however, was abolished in both reports, and with a high-salt diet, K^+^ excretion followed the timing of food intake. This result suggests that the timing of food intake may be a key factor for K^+^ excretion. The definite mechanism is unknown; however, changes in the urinary K^+^ rhythm may have resulted in a change in response to the acute K^+^ load. Furthermore, it has been reported that the peak time of corticosterone secretion moved to the time when feeding was started [41], and that inactive phase pNCC was elevated by corticosterone [42]. The change in corticosterone secretion from inactive time-restricted feeding could have resulted in changes in the diurnal rhythm of the pNCC, and the response to the acute potassium load may have changed as well. Furthermore, feeding rhythm is attenuated in *Clock* mutant mice [43]. As the reversed feeding time reversed the urinary Na^+^ and K^+^ excretion difference from the acute salt load, the disrupted feeding rhythm in *Clock* mutant mice may be the reason for the loss in timing difference. Therefore, time-restricted feeding may restore the circadian arrhythmicity of Na^+^ and K^+^ excretion in *Clock* mutant mice because night-time restricted feeding recovered the rhythmicity of *Bmal1* and *Rev-erva* gene expression in colon tissue of *Clock* mutant mice [44]. In addition, to understand the detailed mechanism of the current results, we should analyze the circadian rhythm of Na^+^/Cl^−^ co-transporter and ENaC activity, as well as blood pressure/heart rate after NaCl/KCl oral administration at different timing in future experiments.

## 5. Conclusions

In conclusion, the intake timing of Na^+^ and K^+^, ZT18, the mid-point of the active phase, is better to achieve natriuretic and kaliuretic effects in mice.

## Data Availability

The original contributions presented in the study are included in the article, further inquiries can be directed to the corresponding author.
